# Detection of Lattice Deformation and Recovery in Epsilon Phase Silver-Tin Alloys

**DOI:** 10.6028/jres.068A.030

**Published:** 1964-06-01

**Authors:** R. M. Waterstrat, G. E. Hicho

## Abstract

A line-broadening effect has been observed in x-ray diffraction patterns from powdered epsilon phase silver-tin base alloys which have been subjected to cold work. The line breadth is reduced when the alloy ingots have received a prior homogenization treatment at 400° C and is also reduced when cold-worked alloy particles are annealed at 100° C. It is shown that the reduction in line breadth which occurs at 100° C is the result of a recovery process which significantly reduces the amount of internal deformation present in the alloy particles. The presence of internal deformation in these cold-worked alloy particles has also been associated with a dimensional expansion which occurs when the silver-tin particles are reacted with mercury at room temperature. Spherical alloy powders produced by an atomization process were found to be relatively free of internal deformation but were not chemically homogeneous. Metallographic and x-ray diffraction data indicated, however, that the spherical alloy particles can be homogenized by a high temperature heat treatment.

## 1. Introduction

X-ray diffraction measurements of line broadening may be used to detect lattice strains and other defects in cold-worked metals. As yet no such measurements have been reported for epsilon phase silver-tin alloys. The purpose of the present investigation was to observe the effects of cold working and also to follow the recovery process which may occur during the annealing of these alloys. The occurrence of such a recovery process at room temperature has been suggested by reports of “aging” phenomena in commercial silver-tin alloys used in dentistry which have been stored for long periods of time [1, 2].^1^ Since recovery may occur even at room temperature it is important that the line-broadening measurements be made on alloys which have not been stored for any appreciable time subsequent to their deformation by cold working. This was made possible through the cooperation of W. D. Kimmel of the L. D. Caulk Co. and W. S. Crowell of the S. S. White Manufacturing Co. who provided the silver- tin alloy particles which had been recently cold worked by machining the alloy ingots. Silver-tin alloy powders have also been prepared using an “atomization” process in which no cold working is involved [3]. These alloy particles are nearly spherical in shape and may be separated into portions consisting of various ranges of particle diameters. It is of interest to determine whether these spherical particles are relatively free of internal microstresses as one might expect from the nature of the “atomization” process. The spherical silver-tin alloy particles were, therefore, examined both by x-ray diffraction and by metallographic methods.

When silver-tin particles are reacted with limited amounts of mercury, a hardening of the mixture occurs which is accompanied by dimensional changes [1, 2]. It has been observed that amalgams prepared from freshly cut alloy particles harden faster and show a greater expansion during hardening than amalgams prepared from particles which have received an “aging” heat treatment in order to stabilize their behavior at room temperature over long periods of time [1, 2]. These observations suggest that the deformation introduced during the preparation of the alloy particles affects the subsequent behavior of these alloys during their reaction with mercury.

The observed correlation between the use of freshly cut alloy particles and the occurrence of a dimensional expansion when these alloys are reacted with mercury might be interpreted as being associated with a recovery process in which internal strains are relieved as mercury diffuses into the silver-tin alloy particles and thereby lowers the activation energy required to initiate such a recovery process. Since the recovery of these strains may also be thermally activated it is possible that a specimen containing no mercury would also exhibit a nonrecoverable change in dimensions during heating. This effect would be superimposed on the normal thermal expansion. Such an effect has been observed and must, therefore, be associated with the behavior of the silver-tin alloy alone rather than with the products of a reaction with mercury.

## 2. Experimental Procedures

The compositions of the alloys used in this investigation are given in table 1. The alloys were obtained in the form of fine shavings which passed a 180-mesh screen. These shavings were produced from cast ingots by machining them in a lathe.

It was considered desirable also to examine an ingot in the “as-cast” condition; one of the alloys was therefore cast in the shape of a rectangular plate 1 in. wide by 2 in. long by ⅛ in. thick. This casting was produced in an investment-type mold under conditions similar to those used in casting the other ingots. The rectangular shape was necessary due to the limitations imposed on sample geometry by the sample holder in the diffractometer and because a flat surface which has not been subjected to a machining operation is needed.

**Figure 1:**
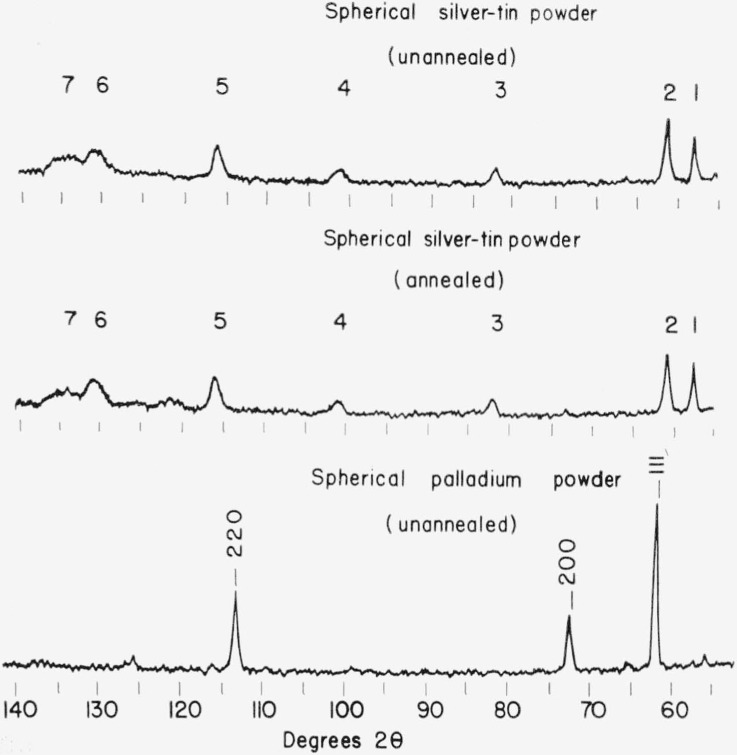
X-ray diffraction patterns for annealed and unannealed spherical silver-tin alloy powders and for a spherical palladium powder.

The spherical alloy powders were prepared by the Federal Mogul Division^2^ and the details of their analysis and preparation have been reported previously [3]. The spherical silver-tin alloy had been stored at room temperature for over one year, however, and it was suspected that a considerable amount of recovery might have occurred during this prolonged period of time. It is desirable to know whether the “atomization” process can be used in preparing metal particles which are relatively strain free. This information was obtained by examining a sample consisting of spherical palladium particles which had been prepared by the same “atomization” process and which had been stored for only four months at room temperature. The x-ray pattern obtained from the spherical palladium particles is given in figure 1 together with x-ray patterns obtained from spherical silver-tin alloy particles in both the “as received” condition and after an annealing treatment consisting of heating for 8 hr at 100 °C in a vacuum.

**Figure 2:**
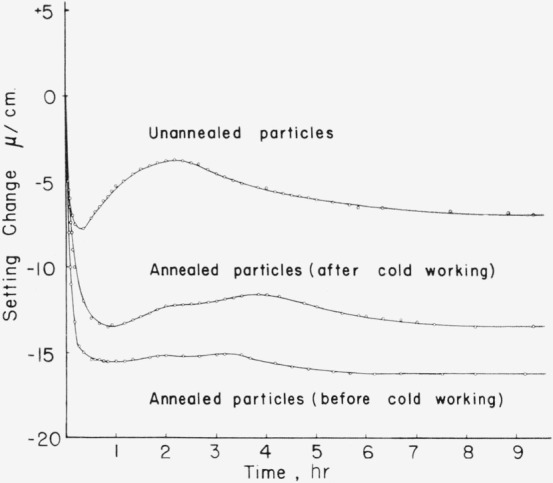
Dimensional changes during hardening for amalgams prepared from a commercial silver-tin alloy (Alloy 112–9).

**Figure 3:**
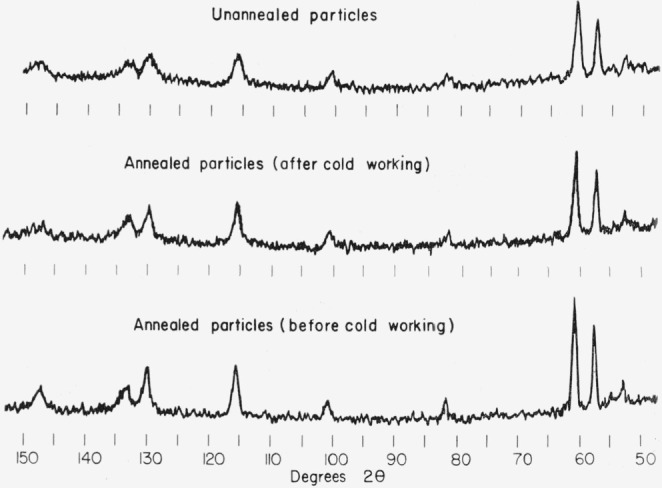
X-ray diffraction patterns of annealed and of cold-worked commercial silver-tin alloy (Alloy 112-9).

Silver-tin alloy particles which had received different annealing treatments or varying amounts of deformation at room temperature were mixed with mercury to form amalgam alloys which hardened in several hours at a temperature of 37 °C. Dimensional changes which occurred during the hardening reaction were measured with an optical interferometer and some typical results are presented in figure 2. X-ray diffraction patterns were obtained from the same samples of silver-tin alloy particles which were used in preparing these amalgams and the equivalent patterns are shown in figure 3. Each amalgam specimen contained 0.6 g of silver-tin alloy particles and was mixed with 0.9 g of mercury in a commercial amalgamator which shakes the mixture vigorously for about 60 sec in order to wet the particles and initiate the hardening reaction. The mixture was then packed into cylindrical steel molds using a steel instrument to pack the mixture firmly. Some excess mercury was removed during this operation. After the amalgam specimens had been packed in the steel mold, they were of a standard size and shape suitable for the measurements of dimensional changes. They were then promptly removed from the steel mold and immediately placed in the interferometer in order that 1 lie dimensional changes might be observed as the hardening reaction progressed.

The x-ray diffraction patterns were obtained using a General Electric XRD–5 diffractometer. The specimens were prepared by sprinkling the alloy powder uniformly over the surface of a glass slide and then spraying the surface of the powder with a thin coat of Krylon acrylic resin to hold it in place. This procedure avoids the introduction of any deformation during the process of specimen preparation. All data were obtained using filtered chromium radiation and the line widths were measured directly from the recorder chart. Each peak was measured at a position corresponding to one half its total peak height. Instrumental broadening of lines was not considered since all patterns were obtained under identical conditions and since only the differences in line broadening rather than the absolute breadth of the lines were used in establishing conclusions.

In order to identify the epsilon phase, it is necessary to make measurements at high angles which are inaccessible to the diffractometer. These measurements were obtained using a Phillips 114.59 mm diam powder camera with filtered chromium radiation.

The metallographic specimens were polished using standard techniques except that diamond polishing followed by magnesium oxide polishing was used in the final stages. Two different etching methods were used. The first method is essentially the one used by Crowell [4]. This etch delineates the grain boundaries and reveals twinning but shows little color contrast between the epsilon phase and retained zeta phase. Therefore, in alloys containing retained zeta phase a duplex etch was used which has been described by Wing [5] and was developed primarily for use with dental amalgams. With this etch the zeta phase particles were seen to have a darker color while the epsilon phase remained unattacked.

## 3. Experimental Results

**Figure 4:**
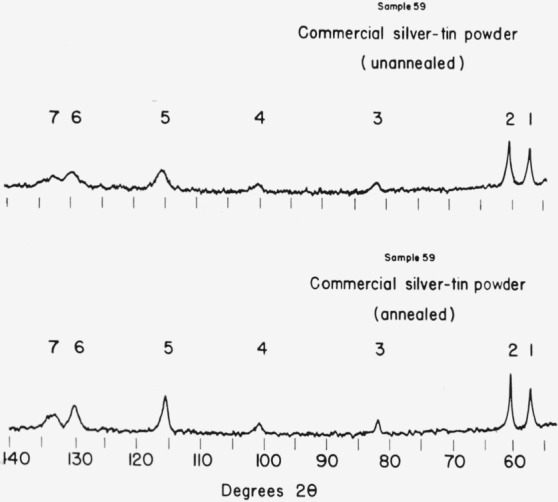
X-ray diffraction patterns for annealed and unannealed commercial silver-tin alloy powders.

A comparison of the results of line-width measurements for annealed and unannealed epsilon phase alloys is given in table 2. The annealing treatments produced a marked reduction in the line widths as shown in figures 3 and 4. The angular dependence of the line broadening is irregular but the higher angle lines are broadened to a greater extent than low angle lines as is usually the case in x-ray patterns obtained from cold-worked metals.

In the commercial sii ver-tin alloys annealed at 100 °C, the use of high-temperature annealing treatments at about 400 °C prior to cold working may sometimes result in x-ray patterns having slightly narrower lines than would be obtained in similar samples which had not received a prior high-temperature anneal. This result suggests the presence of composition gradients and chemical inhomogeneity which is removed by interatomic diffusion at 400 °C but is not appreciably affected at 100 °C over short periods of time.

The x-ray pattern obtained from the flat rectangular ingot of silver-tin alloy in the “as-cast” condition contains broad lines which become narrower after annealing at 100 °C. Annealing this ingot at 400 °C produces a much greater reduction in line width. This indicates the presence of chemical inhomogeneity in the cast ingot and probably also some internal strains resulting from nonuniform stress distribution imposed by thermal contractions during cooling.

X-ray patterns obtained from spherical alloy particles are unaffected by low temperature annealing treatments (fig. 1). The breadth of the lines may be reduced, however, by high-temperature annealing at 400 °C. Apparently considerable chemical inhomogeneity is retained in these particles but they do not contain the large amount of internal strains which are present in particles which have been cold-worked during their preparation. The spherical palladium particles are also quite free of internal strains as indicated by the sharp peaks in the x-ray pattern shown in figure 1. It is unlikely that appreciable recovery could occur in pure palladium at room temperature in four months and therefore the particles must have been quite free of internal strains even immediately following their initial preparation by the “atomization” process. Since compositional segregation cannot occur in a pure metal, the x-ray patterns for pure palladium spherical particles are sharp, whereas x-ray patterns from spherical silver-tin particles contain broader lines as a result of this type of segregation.

**Figure 5:**
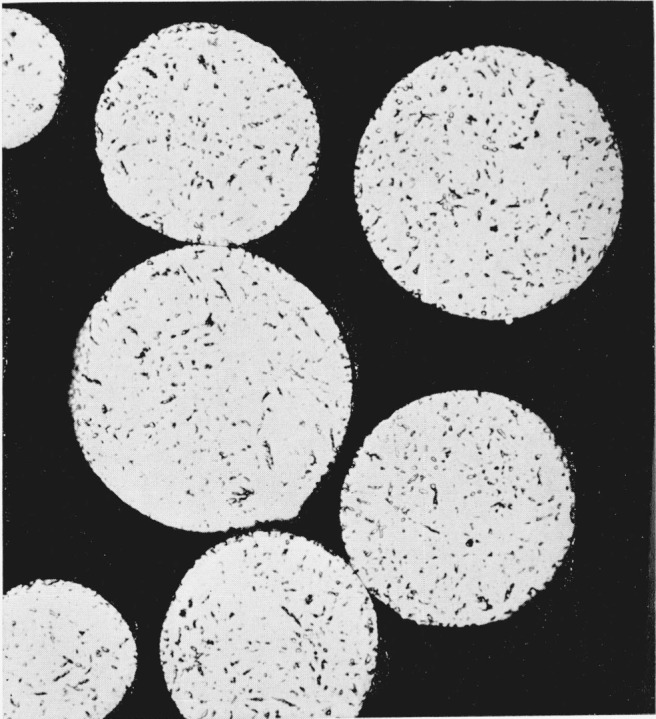
Microstructure of spherical silver-tin alloy particles annealed for 1 hr at 100 ° C. Epsilon phase matrix containing a dispersion of small globules of zeta phase 500 ×.

**Figure 6:**
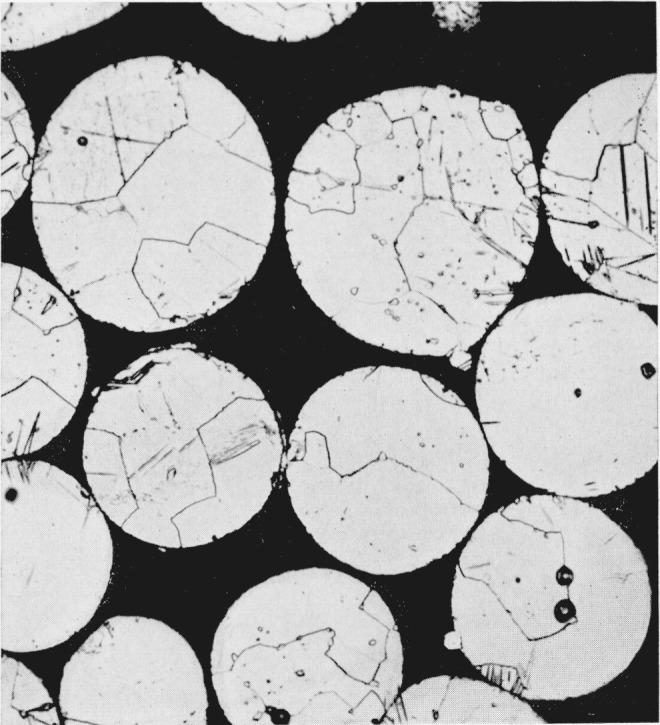
Microstructure of spherical silver-tin alloy particles annealed for 24 hr at 400 °C. Large grains of epsilon phase containing twins. 500 ×.

Metallographic observations support such an interpretation of line broadening effects in silver-tin spherical particles. Figure 5 shows the microstructure of the spherical silver-tin particles after annealing at only 100 °C. The matrix is epsilon phase with very small globules of a second phase distributed uniformly throughout the matrix. The second phase is removed by annealing at 400 °C as shown in figure 6 and the microstructure is then essentially single phase. The individual grains are now quite large relative to the dimensions of each particle and they contain numerous twins. An x-ray diffraction pattern was obtained for this specimen in the Phillips camera and this pattern was completely indexed on the basis of an orthorhombic unit cell similar to the one proposed by Nial, Almin, and Westgren [7] for the epsilon phase, Ag_3_Sn. The unit cell dimensions obtained for the spherical alloy were *a*_0_=2.983 Å, *b*_0_=5.132 Å, and *c*_0_=4.760 Å.

A correlation is established between dimensional expansion in silver-tin amalgams during the hardening reaction and the presence of cold work in the silver-tin alloy particles by consideration of figures 2 and 3. This correlation is in accord with previous observations [1, 2] and is particularly significant in the present study since particles which had been annealed in order to obtain strain-free and homogeneous structures have now been cold worked by grinding in a mortar and pestle and subsequently checked for x-ray line broadening as well as for dimensional expansion during hardening of their amalgams.

An increased dimensional expansion and a broadening of x-ray lines are produced by cold working the previously strain-free and homogeneous particles as can be seen in figures 2 and 3. The question now arises whether the expansion is due to products of the reaction with mercury or whether much of this expansion results from the presence of internal strains in the silver-tin alloy particles. An experiment was performed in order to help resolve this question. If the presence of deformation in the silver-tin alloy particles is responsible for a significant amount of the expansion obtained during amalgamation, then presumably the internal strains associated with this deformation are recoverable by the diffusion of mercury into the silver-tin alloy particles. These internal strains might, therefore, also be recoverable in silver-tin alloys which contain no mercury by heating them to a temperature at which recovery may occur by a thermally activated process. Accordingly a commercial silver-tin alloy powder was compressed in a cylindrical steel mold at room temperature under a pressure of approximately 140,000 psi. This pressure was sufficient to produce severe plastic deformation. The small specimen was then heated uniformly in a specially designed high- temperature interferometer in which dimensional changes are automatically recorded during both heating and cooling of the sample [6]. An expansion was observed during heating which was independent of the normal thermal expansion and superimposed upon it. The amount of expansion produced seemed to depend on both time and temperature. This expansion was permanent and could not be removed by cooling to room temperature; in other words the specimen had a permanent increase in length after returning to room temperature. Further expansion occurred in a subsequent reheating when the maximum temperature reached in the previous heating was exceeded. These results will be reported in detail in a subsequent publication [6]. The total expansion obtained in heating this specimen to 150 °C was about 10 *μ*/cm; a value which is certainly significant when compared to the expansions shown in figure 2 for the silver-tin amalgams.

## 4. Discussion

The broadening of x-ray diffraction lines may be caused by compositional inhomogeneity in an alloy as a result of segregation which occurs as the alloy cools from the liquid state. Examination of the binary silver-tin constitution diagram [8] in the composition range corresponding approximately to that of the alloys used in this study shows that a pronounced tendency toward segregation would be expected. The observed data confirm this expectation but also indicate that all of the observed line broadening cannot be due to this effect since significant reductions in line widths occur at a temperature of 100 °C which is too low to permit the long range interatomic diffusion necessary to remove chemical segregation. Figures 5 and 6 support such a conclusion since the anneal at 100 °C did not remove the second phase while a subsequent anneal at 400 °C was quite effective in dissolving it.

X-ray diffraction lines are broadened by the presence of small particles, and coherency strains could also have some effect on line broadening when more than one phase is present. In a homogeneous, single phase alloy, however, changes in the width of x-ray diffraction lines are due primarily to the introduction of internal defects which disturb or modify the perfect periodicity of the atomic arrangement [9]. These internal defects may be introduced either during the growth of the crystal or by subsequent mechanical working at a temperature which is too low to permit their removal by thermally activated processes. It has been shown in this study that line broadening is produced in homogeneous, strain-free epsilon phase alloy particles when they are subjected to the cold working effects of grinding in a mortar and pestle. Prior to receiving such treatment these particles produced x-ray patterns in which the lines were very narrow and sharp (fig. 3). This implies that any defects which may have been present in these alloys as a result of crystal growth processes are not responsible for the observed changes in line width and that the observed line broadening is the result of cold working.

The nature of the defects produced in cold-worked metals is a subject of considerable controversy. It has been suggested that the effects on line width are due to the presence of internal microstresses [9, 10]. The line broadening has also been attributed to the presence of small coherently diffracting [9, 10] domains produced by cold working. The angular dependence of line broadening would differ for each of these two mechanisms but complications which produce an irregular angular dependence in the present study also prevent any conclusion regarding the nature of the cold-worked metal.

These complications arise from the presence of a second phase in many of the alloys used. Lines from this second phase superimpose on the epsilon phase lines thus preventing measurements of the line broadening originating only in the epsilon phase. Even in the single phase alloys, however, a quantitative analysis of the line broadening depends to a great extent on a detailed knowledge of the atomic structure including such information as the nature and degree of ordering which is as yet unavailable for the epsilon phase alloys.

It has been shown in this study that the principal constitutent of these alloys has a crystal structure which is probably isomorphous with the structure of the binary silver-tin epsilon phase (Ag_3_Sn) as reported by Nial, Almin, and Westgren [7]. Some of the silver atoms, however, are presumably replaced by copper atoms in such a way as to preserve the “ideal” stoichiometric formula (Ag, Cu)_3_ Sn. When cooled from the liquid state these alloys contain varying amounts of a second phase which is probably the zeta phase. The zeta phase has a close- packed hexagonal crystal structure [7, 8]. The crystal structure of the epsilon phase is similar to that of the zeta phase except for a small distortion which might be associated with a different ordering of the silver and tin atoms. The orthorhombic distortion produces a slight “splitting” of the x-ray diffraction lines for the close-packed hexagonal structure and this “splitting” is detectable principally at higher angles. The presence of small quantities of zeta phase in an epsilon phase silver-tin alloy would, therefore, be difficult to detect in x-ray diffraction patterns of these alloys. Metallographic studies, however, have been successful in identifying the major constituent of these alloys as the epsilon phase.

The fine dispersion of zeta phase globules in the spherical silver-tin particles (fig. 5) is probably attributable to the rapid cooling rates involved in the “atomization” process which tend to suppress the peritectic reaction involved in the formation of the epsilon phase. Annealing these particles at a high temperature (400 °C) which lies below the temperature of the peritectic reaction produces an equilibrium structure (fig. 6) which is essentially that of a single-phase epsilon alloy. This is confirmed by the x-ray diffraction measurements as well as by the metallographic observations. In figures 2 and 3 an interesting correlation is established between the presence of cold work in silver-tin alloy particles and the observed dimensional changes as these particles react with mercury. This effect receives further clarification in the experiment which demonstrated that silver-tin alloys subjected to considerable plastic deformation in compression will subsequently exhibit a permanent nonrecoverable expansion when heated.

The evidence suggests that internal strains or other lattice defects produced by cold working these alloys may be relieved through a recovery process which occurs over long periods of time at room temperature and is accelerated at higher temperatures. This recovery process is probably thermally activated. When mercury is dissolved in the silver-tin alloys it apparently lowers the activation energy required for this recovery process perhaps by lowering the melting point and weakening the interatomic binding forces. When this occurs the thermal energy available at room temperature is sufficient to activate the recovery process.

## 5. Conclusions

The results of this study lead to the following conclusions:

1. X-ray diffraction techniques may be used to detect lattice deformation in epsilon phase silver-tin alloys which have been recently subjected to cold working. Alloys of this type are commonly used in preparing dental amalgams and such a technique would, therefore, be useful in evaluating their properties.
2. The internal deformation may be removed through a recovery process which occurs when the alloys are heated to higher temperatures. The recovery process may also occur at room temperature when considerable time is available or when mercury is allowed to dissolve in the alloy particles.
3. The “atomization” process used to prepare spherical alloy particles results in the production of particles which are relatively strain free in the size ranges tested. Compositional segregation is not prevented but occurs as a fine, uniform dispersion of a second phase. The alloy particles may subsequently be homogenized by a heat treatment at high temperatures.
4. The x-ray pattern of the epsilon phase in these silver-tin base alloys has been identified as that of an orthorhombic crystal structure similar to the one proposed by Nial, Almin, and Westgren for the phase Ag_3_Sn.
5. It is likely that significant dimensional changes occur when silver-tin alloy particles react with mercury as a result of the release of internal strains in the cold-worked alloy particles.

## Figures and Tables

**Table. 1:** Approximate chemical compositions of alloys

Alloy	Composition—% by weight			
	Silver	Tin	Copper	Zinc
				
Spherical alloy*	70.6	26.1	2.5	0.4
50	Not determined			
51	68.5	26.0	4.8	.7
52	72.9	26.1	1.0	.0
53	72.2	25.8	1.0	1.0
54	69.6	26.4	3.8	0.2
55	69.7	26.5	3.8	.0
59	72.2	25.8	1.0	1.0
60	69.6	26.4	3.8	0.2
61	69.7	26.5	3.8	.0
112–9	70.9	25.7	2.4	1.0

* Composition was obtained by chemical analysis [3].

**Table 2:** Results of measurements of line widths in annealed and in unannealed silver-tin alloys

Sample identification	High-temperature anneal	Low-temperature anneal	Observed relative half widths								
			2*θ*	57°	61°	82°	101°	115°	130°	133°	148°
			*hkl*		111	112	200	113	202	221	
				002	021	022	130	023	132	041	004
											
50A	None	None		6.0	7.0	9.0	17.0	11.5	14.0	18.0	
50B	None	100 °C—1 hr		3.5	4.0	6.0	10.0	6.0	10.0	14.0	
51A	None	None		5.0	6.0	9.0	12.0	14.0	17.0	18.0	
51B	None	100 °C—1 hr		4.5	5.5	7.0	9.5	7.5	10.0	15.0	
51C	None	100 °C— 3 hr		4.0	5.0	6.0	9.0	6.0	13.0	12.0	
52A	432 °C—72 hr	None		4.0	4.0	4.0	12.0	10.0	14.5	16.0	
52B	432 °C—72 hr	100 °C—1 hr		3.5	4.5	5.0	5.0	7.0	9.0	12.0	
52C	432 °C—72 hr	100 °C—3 hr		3.0	4.0	5.0	7.0	6.0	10.0	14.0	
53A	432 °C—72 hr	None		5.0	4.0	7.0	7.5	13.0	19.0	19.0	
53B	432 °C—72 hr	100 °C—1 hr		4.0	4.0	5.0	7.0	7.0	11.0	15.0	
53C	432 °C—72 hr	100 °C—3 hr		4.0	4.0	5.0	8.0	5.0	12.0	13.0	
54A	None	None		4.0	5.0	6.0	13.0	10.0	17.0	21.0	
54B	None	100 °C—1 hr		3.5	4.5	6.0	10.0	7.0	8.0	17.5	
54C	None	100 °C—3 hr		4.0	4.0	5.0	7.0	9.0	9.0	15.0	
55A	Nono	None		5.0	6.0	5.0	8.0	10.0	15.0	21.0	
55B	None	100 °C—1 hr		3.0	4.0	5.0	7.0	5.0	9.5	12.0	
55C	None	100 °C—3 hr		3.0	4.0	5.0	8.0	6.0	8.0	13.0	
59A	None	None		5.0	5.0	10.0	10.0	13.0	15.0	22.0	
59B	None	100 °C—1 hr		3.0	3.5	4.0	9.0	5.0	7.5	14.0	
60A	432 °C—72 hr	None		6.0	6.0	11.0	11.5	10.0	15.0	25.0	
60B	432 °C—72 hr	100 °C—1 hr		3.5	4.5	3.0	8.0	6.0	11.0	17.0	
61A	432 °C—72 hr	None		5.0	5.5	9.0	18.0	11.5	13.0	31.0	
61B	432 °C—72 hr	100 °C—1 hr		4.0	5.0	10.0	10.0	6.0	12.0	11.0	
112-9A	None	100 °C—3 hr		4.0	4.5	11.0	12.0	10.0	15.0	21.0	16.0
112-9B	400 °C—16 hr	None (cold worked)		4.0	5.5	11.0	11.5	13.0	16.0	20.0	20.0
112-9C	400 °C—16 hr	100 °C—3 hr		3.0	4.0	5.0	8.5	7.0	9.0	13.5	11.0
112-9D	400 °C—16 hr	100 °C—3 hr and then cold worked		4.0	4.5	8.0	13.0	8.0	9.5	14.0	20.0
Average for unannealed particles	……	……	……	5.0	5.8	8.8	12.6	11.6	15.3	22.0	20.0
Average for all particles annealed at 100 °C	……	……	……	3.6	4.3	5.8	8.4	6.6	10.3	14.3	13.5
Flat casting 1A	None	None		6.5	8.5	11.0	15.0	17.0	22.0	24.0	
Flat casting 1B	None	100 °C—24 hr		5.0	7.0	8.5	8.5	10.5	23.0	17.0	
Flat casting 1C	400 °C—24 hr	None		2.5	5.5	5.0	4.0	6.0	12.0	8.0	

## Footnotes

1 Figures in brackets indicate the literature references at the end of this paper.

2 Federal Mogul Division, Federal-Mogul-Bower Bearings, Inc., Ann Arbor, Mich.
